# Al_0.88_Cu_0.94_Fe_0.18_

**DOI:** 10.1107/S2414314623008702

**Published:** 2023-10-12

**Authors:** Yibo Liu, Huizi Liu, Bin Wen, Changzeng Fan

**Affiliations:** a State Key Laboratory of Metastable Materials Science and Technology, Yanshan University, Qinhuangdao 066004, People’s Republic of China; b Hebei Key Lab for Optimizing Metal Product Technology and Performance, Yanshan University, Qinhuangdao, Hebei 066004, People’s Republic of China; University of Aberdeen, United Kingdom

**Keywords:** crystal structure, high-temperature sinter­ing, *β* phase, Al–Cu–Fe system

## Abstract

The inter­metallic phase with composition Al_0.88_Cu_0.94_Fe_0.18_ was synthesized by high-temperature sinter­ing of a mixture with initial chemical composition Al_78_Cu_48_Fe_13_. Al_0.88_Cu_0.94_Fe_0.18_ adopts the CsCl structure type in space-group *Pm*  *m*. The structure analysis revealed that one site is co-occupied by Al and Cu with a ratio of 0.88 (5):0.12 (5) and the other is co-occupied by Fe and Cu with a ratio of 0.2 (4):0.8 (4). The Al/Cu⋯Fe/Cu separation is 2.5465 (13) Å.

## Abstract 2

The inter­metallic phase with composition Al_0.88_Cu_0.94_Fe_0.18_ was synthesized by high-temperature sinter­ing of a mixture with initial chemical composition Al_78_Cu_48_Fe_13_. Al_0.88_Cu_0.94_Fe_0.18_ adopts the CsCl structure type in space-group *Pm* 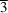 *m*.

## Graphical abstract

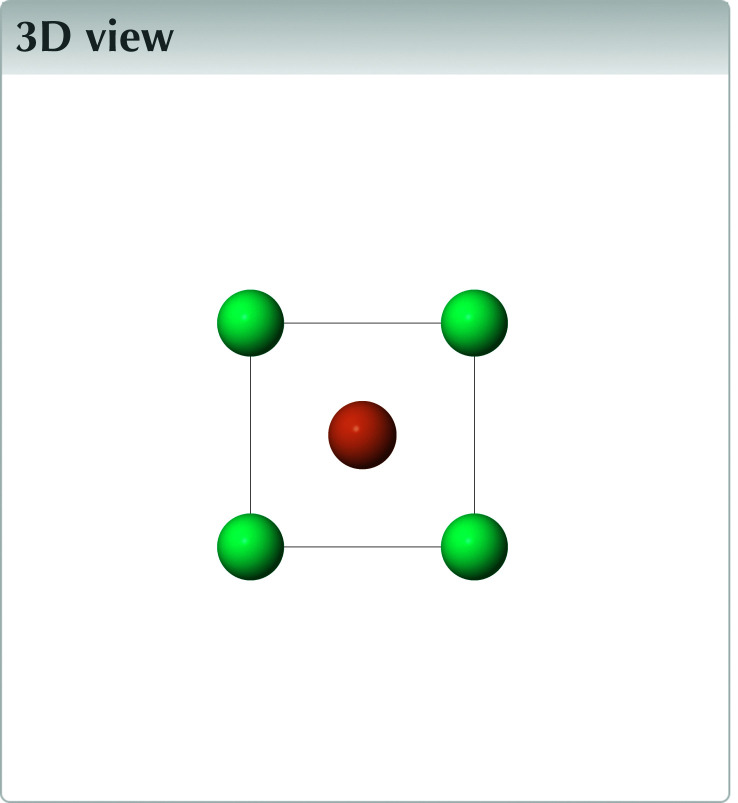

## Structure description

Phases in the ternary Al–Cu–Fe alloy system often have complex crystal structures as well as quasicrystals (QC). For example, an aperiodic diffraction pattern was observed for the alloy with composition Al_63_Cu_24_Fe_13_, exhibiting tenfold rotation symmetry and characterized as a quasi-crystalline phase, as revealed by the first natural quasicrystal (Bindi *et al.*, 2011 ▸). The present phase Al_0.88_Cu_0.94_Fe_0.18_ belongs to the *β*-phase in the Al–Cu–Fe system, which is similar to that of the B2-FeAl phase (Rosas & Perez,1998 ▸). Meyer *et al.* (2007 ▸) suggested that the *β*-phase has a b.c.c. crystal structure, and the lattice parameter of *β*-Al_50_Cu_20_Fe_30_ was *a* = 2.925 Å as determined by X-ray diffraction. Kalmykov *et al.* (2009 ▸) studied the Al–Cu–Fe phase diagram at 853 K, and considered that the lattice parameter of the *β*-AlCuFe phase increased with the increase of Cu content. The lowest copper content of the *β*-phase is 7.3 at.% corresponding to a lattice parameter of *a =* 2.9171 Å, while the *β*-phase with the highest copper content of 45.5 at.% has *a =* 2.9390 Å. Shalaeva & Prekul (2011 ▸) studied two kinds of *β*-phases with nominal composition of Al_50_Cu_33_Fe_17_, namely the *β*_1_- and *β*_2_-phases. The lattice parameters of the two phases were found to be 2.939 and 2.969 Å, respectively, by X-ray diffraction, and the average compositions of the two phases were Al_51.5_Fe_19_Cu_29.5_ and Al_48.5_Fe_13_Cu_38.5_, respectively, by the electron-probe method. It should be noted that only the lattice parameters of the *β*-phase have been given in the aforementioned studies while an exact crystal structural model has not been provided. According to the Springer Materials database, there are several crystal-structure models for the *β*-phase in previous studies; however, such a given structure model only represents a possibility inferred from the composition rather than a refined one.

In the present study, the crystal structure model for the *β*-phase in the Al–Cu–Fe system has been refined on basis of single-crystal X-ray diffraction data. This phase has similar lattice parameters to the previously reported *β*-phase. Its chemical composition was refined to be Al_0.88_Cu_0.94_Fe_0.18_, in accordance with the complementary EDX results (see Table S1 of the supporting information).

**Figure 1:**
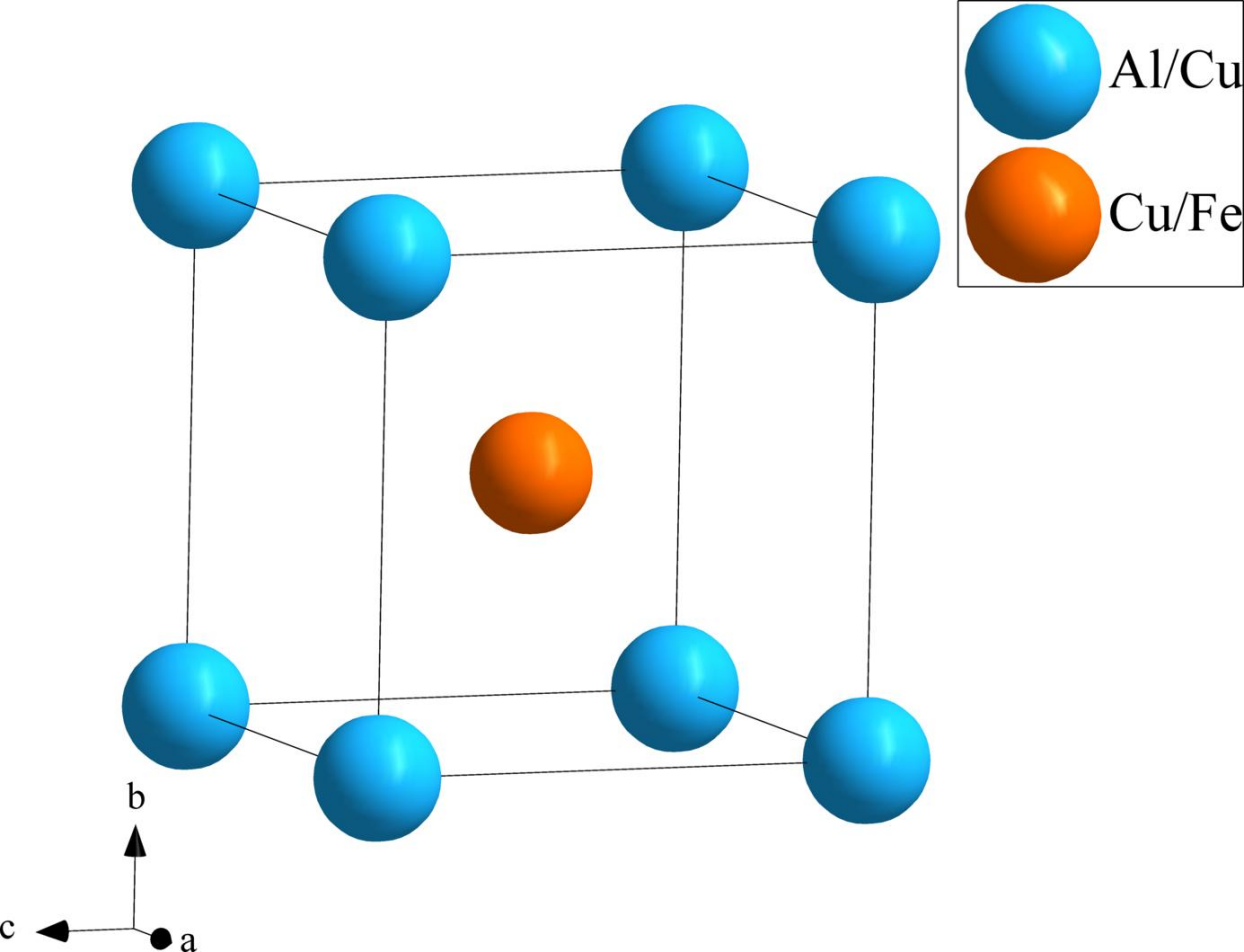
The crystal structure of Al_0.88_Cu_0.94_Fe_0.18._

**Figure 2:**
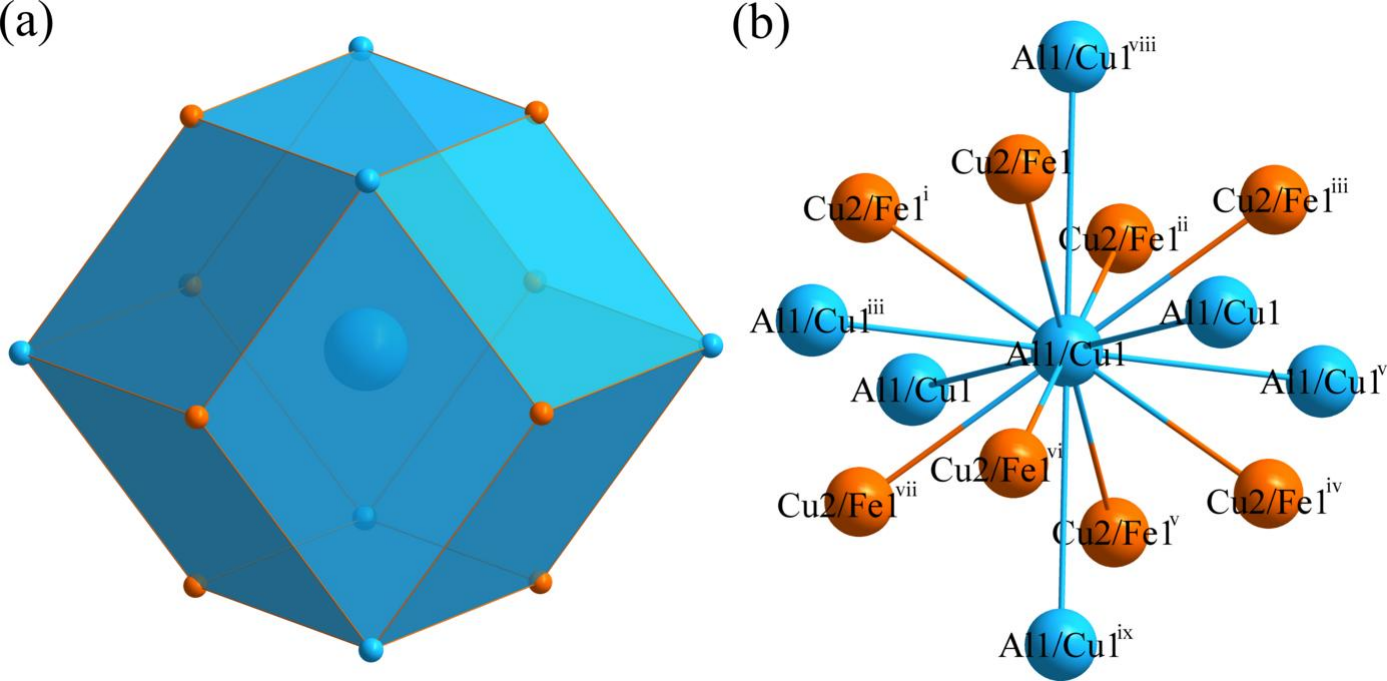
(*a*) The dodeca­hedron formed around the Al1/Cu1 atom at the 1*a* site; (*b*) the environment of the Al1/Cu1 atom with displacement ellipsoids given at the 99% probability level. [Symmetry codes: (i) *x* − 1, *y* − 1, *z* − 1; (ii) *x* − 1, *y*, *z*; (iii) *x*, *y* − 1, *z*; (iv) *x* − 1, *y* − 1, *z*; (v) *x*, *y*, *z* − 1; (vi) *x* − 1, *y*, *z* − 1; (vii) *x*, *y* − 1, *z* − 1; (viii) *x*, *y*, *z* + 1; (ix) *x*, *y* + 1, *z*.]

Fig. 1 ▸ shows the distribution of the atoms in the unit cell of Al_0.88_Cu_0.94_Fe_0.18_. The environments of the Al1/Cu1 and Cu2/Fe1 sites are shown in Figs. 2 ▸ and 3 ▸, respectively. The Al1/Cu1 atom at (0, 0, 0) is centered at a dodeca­hedron, which is surrounded by six Al1/Cu1 atoms and eight Cu2/Fe1 atoms; conversely, the Cu2/Fe1 site at (1/2, 1/2, 1/2) is surrounded by eight Al1/Cu1 atoms and six Cu2/Fe1 atoms. The shortest Al1/Cu1 to Cu2/Fe1 separation is 2.5465 (13) Å and the shortest Al1/Cu1 to Al1/Cu1 contact is 2.9405 (15) Å.

## Synthesis and crystallization

The high-purity elements Al (indicated purity 99.95%; 0.7163 g), Cu (indicated purity 99.99%; 1.0372 g) and Fe (indicated purity 99.9%; 0.2485 g) were mixed in the molar ratio 78:48:13 and ground in an agate mortar. The blended powders were placed into a cemented carbide grinding mound of 9.6 mm diameter and pressed at 4 MPa for about 3 min. The obtained cylindrical block was put into a silica glass tube and vacuum-sealed by a home-made sealing machine. The resulting ampoule then was placed in a furnace (SG-XQL1200) and heated up to 1373 K for 2 h with with a heating rate of 10 K min^−1^. The temperature was then reduced to 1073 K for 10 h. Finally, the sample was slowly cooled to room temperature by turning off the furnace power. Suitable pieces of single-crystal grains were broken and selected from the product for single-crystal X-ray diffraction.

## Refinement

Crystal data, data collection and structure refinement details are summarized in Table 1 ▸. All atoms in the unit cell co-occupied the Wykoff positions. Different choices of refinement are listed in Table S2 of the supporting information. The maximum and minimum residual electron densities in the final difference map are located 0.0 Å and 1.01 Å from the atoms Cu1.

## Supplementary Material

Crystal structure: contains datablock(s) I. DOI: 10.1107/S2414314623008702/hb4450sup1.cif

Structure factors: contains datablock(s) I. DOI: 10.1107/S2414314623008702/hb4450Isup2.hkl

ESI. DOI: 10.1107/S2414314623008702/hb4450sup3.docx

CCDC reference: 2299144

Additional supporting information:  crystallographic information; 3D view; checkCIF report

## Figures and Tables

**Figure 3:**
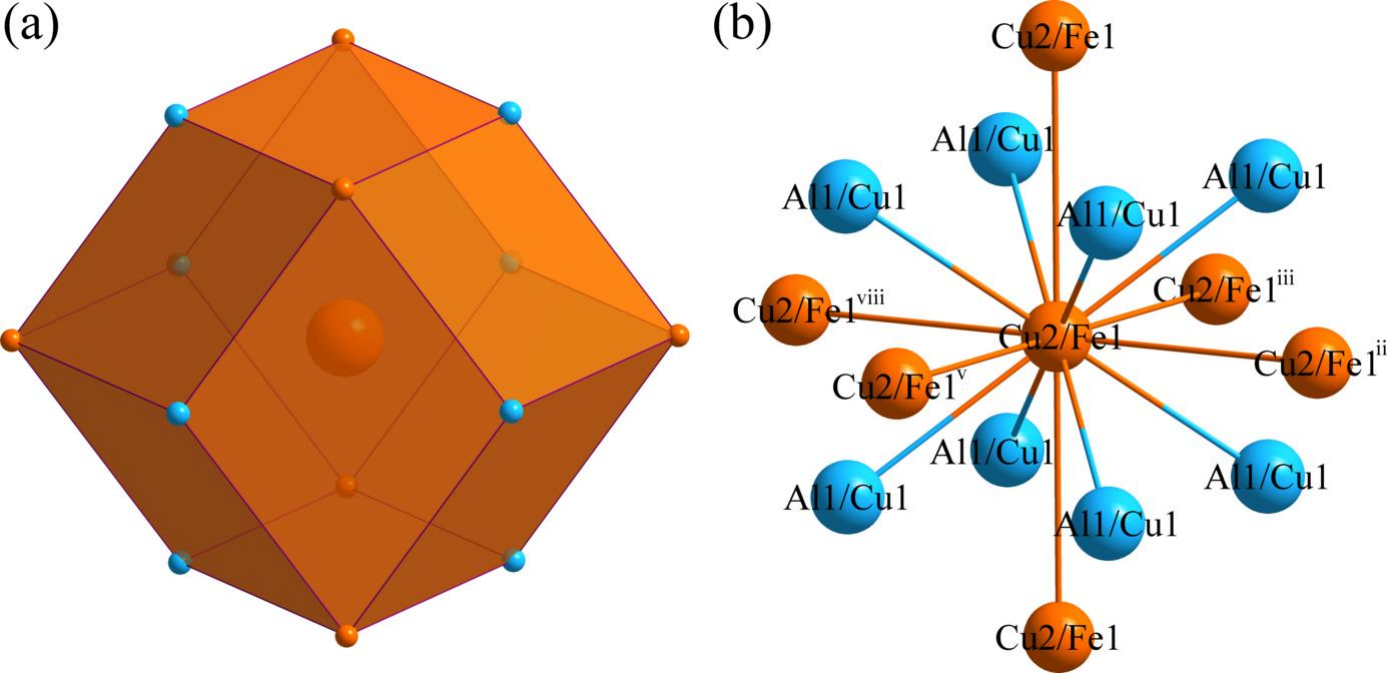
(*a*) The dodeca­hedron formed around the Cu2/Fe1 atom at the 1*b* site; (*b*) the environment of the Cu2/Fe atom with displacement ellipsoids given at the 99% probability level. [Symmetry codes: (ii) *x* − 1, *y*, *z*; (iii) *x*, *y* − 1, *z*; (v) *x*, *y*, *z* − 1; (viii) *x*, *y*, *z* + 1.]

**Table 1:** Experimental details

Crystal data	
Chemical formula	Al_0.88_Cu_0.94_Fe_0.18_
*M*_r_	93.42
Crystal system, space group	Cubic, *P* *m* 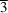 *m*
Temperature (K)	299
*a* (Å)	2.9405 (15)
*V* (Å^3^)	25.43 (4)
*Z*	1
Radiation type	Mo *K*α
μ (mm^−1^)	22.36
Crystal size (mm)	0.14 × 0.12 × 0.12

Data collection	
Diffractometer	Bruker D8 Venture Photon 100 CMOS
Absorption correction	Multi-scan (*SADABS*; Krause et al., 2015 ▸)
*T*_min_, *T*_max_	0.439, 0.746
No. of measured, independent and observed [*I* > 2σ(*I*)] reflections	385, 14, 14
*R*_int_	0.021
(sin θ/λ)_max_ (Å^−1^)	0.636

Refinement	
*R*[*F* ^2^ > 2σ(*F* ^2^)], *wR*(*F* ^2^), *S*	0.013, 0.031, 1.44
No. of reflections	14
No. of parameters	5
Δρ_max_, Δρ_min_ (e Å^−3^)	0.32, −0.48

Computer programs: *APEX3* and *SAINT* (Bruker, 2015 ▸), *SHELXT2014/5* (Sheldrick, 2015*a* ▸), *SHELXL2017/1* (Sheldrick, 2015*b* ▸), *DIAMOND* (Brandenburg & Putz, 2017 ▸) and *publCIF* (Westrip, 2010 ▸).

## full crystallographic data

### Crystal data

**Table:**

Al_0.88_Cu_0.94_Fe_0.18_	Mo *K*α radiation, λ = 0.71073 Å
*M**_r_* = 93.42	Cell parameters from 367 reflections
Cubic, *P**m*3*m*	θ = 6.9–26.9°
*a* = 2.9405 (15) Å	µ = 22.36 mm^−^^1^
*V* = 25.43 (4) Å^3^	*T* = 299 K
*Z* = 1	Lump, drak gray
*F*(000) = 43	0.14 × 0.12 × 0.12 mm
*D*_x_ = 6.101 Mg m^−^^3^	

### Data collection

**Table:**

Bruker D8 Venture Photon 100 CMOS diffractometer	14 reflections with *I* > 2σ(*I*)
phi and ω scans	*R*_int_ = 0.021
Absorption correction: multi-scan (SADABS; Krause et al., 2015)	θ_max_ = 26.9°, θ_min_ = 6.9°
*T*_min_ = 0.439, *T*_max_ = 0.746	*h* = −3→3
385 measured reflections	*k* = −3→3
14 independent reflections	*l* = −3→3

### Refinement

**Table:**

Refinement on *F*^2^	0 restraints
Least-squares matrix: full	Primary atom site location: isomorphous structure methods
*R*[*F*^2^ > 2σ(*F*^2^)] = 0.013	*w* = 1/[σ^2^(*F*_o_^2^) + (0.0246*P*)^2^] where *P* = (*F*_o_^2^ + 2*F*_c_^2^)/3
*wR*(*F*^2^) = 0.031	(Δ/σ)_max_ < 0.001
*S* = 1.44	Δρ_max_ = 0.32 e Å^−^^3^
14 reflections	Δρ_min_ = −0.48 e Å^−^^3^
5 parameters	

### Special details

**Table:**

Geometry. All esds (except the esd in the dihedral angle between two l.s. planes) are estimated using the full covariance matrix. The cell esds are taken into account individually in the estimation of esds in distances, angles and torsion angles; correlations between esds in cell parameters are only used when they are defined by crystal symmetry. An approximate (isotropic) treatment of cell esds is used for estimating esds involving l.s. planes.

### Fractional atomic coordinates and isotropic or equivalent isotropic displacement parameters (Å^2^)

**Table:**

	*x*	*y*	*z*	*U*_iso_*/*U*_eq_	Occ. (<1)
Al1	0.000000	0.000000	0.000000	0.0113 (13)	0.88 (5)
Cu1	0.000000	0.000000	0.000000	0.0113 (13)	0.12 (5)
Fe1	0.500000	0.500000	0.500000	0.0105 (6)	0.2 (4)
Cu2	0.500000	0.500000	0.500000	0.0105 (6)	0.8 (4)

### Atomic displacement parameters (Å^2^)

**Table:**

	*U*^11^	*U*^22^	*U*^33^	*U*^12^	*U*^13^	*U*^23^
Al1	0.0113 (13)	0.0113 (13)	0.0113 (13)	0.000	0.000	0.000
Cu1	0.0113 (13)	0.0113 (13)	0.0113 (13)	0.000	0.000	0.000
Fe1	0.0105 (6)	0.0105 (6)	0.0105 (6)	0.000	0.000	0.000
Cu2	0.0105 (6)	0.0105 (6)	0.0105 (6)	0.000	0.000	0.000

### Geometric parameters (Å, º)

**Table:**

Al1—Fe1^i^	2.5465 (13)	Al1—Al1^v^	2.9405 (15)
Al1—Fe1	2.5465 (13)	Al1—Al1^viii^	2.9405 (15)
Al1—Fe1^ii^	2.5465 (13)	Al1—Al1^ix^	2.9405 (15)
Al1—Fe1^iii^	2.5465 (13)	Cu1—Cu2	2.5465 (13)
Al1—Fe1^iv^	2.5465 (13)	Fe1—Fe1^viii^	2.9405 (15)
Al1—Fe1^v^	2.5465 (13)	Fe1—Fe1^v^	2.9405 (15)
Al1—Fe1^vi^	2.5465 (13)	Fe1—Fe1^iii^	2.9405 (15)
Al1—Fe1^vii^	2.5465 (13)	Fe1—Fe1^ii^	2.9405 (15)
Al1—Al1^iii^	2.9405 (15)		
			
Fe1^i^—Al1—Fe1	180.0	Al1^x^—Fe1—Al1	180.0
Fe1^i^—Al1—Fe1^ii^	109.5	Al1^x^—Fe1—Al1^xi^	109.5
Fe1—Al1—Fe1^ii^	70.529 (1)	Al1—Fe1—Al1^xi^	70.529 (1)
Fe1^i^—Al1—Fe1^iii^	109.5	Al1^x^—Fe1—Al1^ix^	109.5
Fe1—Al1—Fe1^iii^	70.5	Al1—Fe1—Al1^ix^	70.5
Fe1^ii^—Al1—Fe1^iii^	109.5	Al1^xi^—Fe1—Al1^ix^	109.5
Fe1^i^—Al1—Fe1^iv^	70.529 (1)	Al1^x^—Fe1—Al1^xii^	70.529 (1)
Fe1—Al1—Fe1^iv^	109.5	Al1—Fe1—Al1^xii^	109.5
Fe1^ii^—Al1—Fe1^iv^	70.5	Al1^xi^—Fe1—Al1^xii^	70.5
Fe1^iii^—Al1—Fe1^iv^	70.5	Al1^ix^—Fe1—Al1^xii^	70.5
Fe1^i^—Al1—Fe1^v^	109.5	Al1^x^—Fe1—Al1^viii^	109.5
Fe1—Al1—Fe1^v^	70.529 (1)	Al1—Fe1—Al1^viii^	70.529 (1)
Fe1^ii^—Al1—Fe1^v^	109.5	Al1^xi^—Fe1—Al1^viii^	109.5
Fe1^iii^—Al1—Fe1^v^	109.5	Al1^ix^—Fe1—Al1^viii^	109.5
Fe1^iv^—Al1—Fe1^v^	180.0	Al1^xii^—Fe1—Al1^viii^	180.0
Fe1^i^—Al1—Fe1^vi^	70.5	Al1^x^—Fe1—Al1^xiii^	70.5
Fe1—Al1—Fe1^vi^	109.5	Al1—Fe1—Al1^xiii^	109.5
Fe1^ii^—Al1—Fe1^vi^	70.5	Al1^xi^—Fe1—Al1^xiii^	70.5
Fe1^iii^—Al1—Fe1^vi^	180.0	Al1^ix^—Fe1—Al1^xiii^	180.0
Fe1^iv^—Al1—Fe1^vi^	109.5	Al1^xii^—Fe1—Al1^xiii^	109.5
Fe1^v^—Al1—Fe1^vi^	70.5	Al1^viii^—Fe1—Al1^xiii^	70.5
Fe1^i^—Al1—Fe1^vii^	70.529 (1)	Al1^x^—Fe1—Al1^xiv^	70.529 (1)
Fe1—Al1—Fe1^vii^	109.5	Al1—Fe1—Al1^xiv^	109.5
Fe1^ii^—Al1—Fe1^vii^	180.0	Al1^xi^—Fe1—Al1^xiv^	180.0
Fe1^iii^—Al1—Fe1^vii^	70.5	Al1^ix^—Fe1—Al1^xiv^	70.5
Fe1^iv^—Al1—Fe1^vii^	109.5	Al1^xii^—Fe1—Al1^xiv^	109.5
Fe1^v^—Al1—Fe1^vii^	70.5	Al1^viii^—Fe1—Al1^xiv^	70.5
Fe1^vi^—Al1—Fe1^vii^	109.5	Al1^xiii^—Fe1—Al1^xiv^	109.5
Fe1^i^—Al1—Al1^iii^	54.7	Al1^x^—Fe1—Fe1^viii^	54.7
Fe1—Al1—Al1^iii^	125.3	Al1—Fe1—Fe1^viii^	125.3
Fe1^ii^—Al1—Al1^iii^	125.3	Al1^xi^—Fe1—Fe1^viii^	125.3
Fe1^iii^—Al1—Al1^iii^	54.7	Al1^ix^—Fe1—Fe1^viii^	125.3
Fe1^iv^—Al1—Al1^iii^	54.7	Al1^xii^—Fe1—Fe1^viii^	125.3
Fe1^v^—Al1—Al1^iii^	125.3	Al1^viii^—Fe1—Fe1^viii^	54.7
Fe1^vi^—Al1—Al1^iii^	125.3	Al1^xiii^—Fe1—Fe1^viii^	54.7
Fe1^vii^—Al1—Al1^iii^	54.7	Al1^xiv^—Fe1—Fe1^viii^	54.7
Fe1^i^—Al1—Al1^v^	54.7	Al1^x^—Fe1—Fe1^v^	125.3
Fe1—Al1—Al1^v^	125.3	Al1—Fe1—Fe1^v^	54.7
Fe1^ii^—Al1—Al1^v^	125.3	Al1^xi^—Fe1—Fe1^v^	54.7
Fe1^iii^—Al1—Al1^v^	125.3	Al1^ix^—Fe1—Fe1^v^	54.7
Fe1^iv^—Al1—Al1^v^	125.3	Al1^xii^—Fe1—Fe1^v^	54.7
Fe1^v^—Al1—Al1^v^	54.7	Al1^viii^—Fe1—Fe1^v^	125.3
Fe1^vi^—Al1—Al1^v^	54.7	Al1^xiii^—Fe1—Fe1^v^	125.3
Fe1^vii^—Al1—Al1^v^	54.7	Al1^xiv^—Fe1—Fe1^v^	125.3
Al1^iii^—Al1—Al1^v^	90.0	Fe1^viii^—Fe1—Fe1^v^	180.0
Fe1^i^—Al1—Al1^viii^	125.3	Al1^x^—Fe1—Fe1^iii^	125.3
Fe1—Al1—Al1^viii^	54.7	Al1—Fe1—Fe1^iii^	54.7
Fe1^ii^—Al1—Al1^viii^	54.7	Al1^xi^—Fe1—Fe1^iii^	54.7
Fe1^iii^—Al1—Al1^viii^	54.7	Al1^ix^—Fe1—Fe1^iii^	125.3
Fe1^iv^—Al1—Al1^viii^	54.7	Al1^xii^—Fe1—Fe1^iii^	125.3
Fe1^v^—Al1—Al1^viii^	125.3	Al1^viii^—Fe1—Fe1^iii^	54.7
Fe1^vi^—Al1—Al1^viii^	125.3	Al1^xiii^—Fe1—Fe1^iii^	54.7
Fe1^vii^—Al1—Al1^viii^	125.3	Al1^xiv^—Fe1—Fe1^iii^	125.3
Al1^iii^—Al1—Al1^viii^	90.0	Fe1^viii^—Fe1—Fe1^iii^	90.0
Al1^v^—Al1—Al1^viii^	180.0	Fe1^v^—Fe1—Fe1^iii^	90.0
Fe1^i^—Al1—Al1^ix^	125.3	Al1^x^—Fe1—Fe1^ii^	125.3
Fe1—Al1—Al1^ix^	54.7	Al1—Fe1—Fe1^ii^	54.7
Fe1^ii^—Al1—Al1^ix^	54.7	Al1^xi^—Fe1—Fe1^ii^	125.3
Fe1^iii^—Al1—Al1^ix^	125.3	Al1^ix^—Fe1—Fe1^ii^	54.7
Fe1^iv^—Al1—Al1^ix^	125.3	Al1^xii^—Fe1—Fe1^ii^	125.3
Fe1^v^—Al1—Al1^ix^	54.7	Al1^viii^—Fe1—Fe1^ii^	54.7
Fe1^vi^—Al1—Al1^ix^	54.7	Al1^xiii^—Fe1—Fe1^ii^	125.3
Fe1^vii^—Al1—Al1^ix^	125.3	Al1^xiv^—Fe1—Fe1^ii^	54.7
Al1^iii^—Al1—Al1^ix^	180.0	Fe1^viii^—Fe1—Fe1^ii^	90.0
Al1^v^—Al1—Al1^ix^	90.0	Fe1^v^—Fe1—Fe1^ii^	90.0
Al1^viii^—Al1—Al1^ix^	90.0	Fe1^iii^—Fe1—Fe1^ii^	90.0

Symmetry codes: (i) *x*−1, *y*−1, *z*−1; (ii) *x*−1, *y*, *z*; (iii) *x*, *y*−1, *z*; (iv) *x*−1, *y*−1, *z*; (v) *x*, *y*, *z*−1; (vi) *x*−1, *y*, *z*−1; (vii) *x*, *y*−1, *z*−1; (viii) *x*, *y*, *z*+1; (ix) *x*, *y*+1, *z*; (x) *x*+1, *y*+1, *z*+1; (xi) *x*+1, *y*, *z*; (xii) *x*+1, *y*+1, *z*; (xiii) *x*+1, *y*, *z*+1; (xiv) *x*, *y*+1, *z*+1.
