# Supplementary material for: Al0.88Cu0.94Fe0.18
Source: IUCrdata. 2023 Oct 12;8(Pt 10):x230870. doi: 10.1107/S2414314623008702 (PMC10626848; doi:10.1107/S2414314623008702)
Supplement: Supplementary file 3 [file x-08-x230870-sup3.docx]

**SUPPLEMENTARY MATERIALS:**

**Crystal structure of Al_0.88_Cu_0.94_Fe_0.18_**

**Yibo Liu**^a^**, Huizi Liu**^a^, **Changzeng Fan**^a,^***, Bing Zhang**^a^**, Bin Wen**^a^ **and Lifeng Zhang**^b^

^a^ State Key Laboratory of Metastable Materials Science and Technology, Yanshan University,

Qinhuangdao 066004, People’s Republic of China

^b^ School of Mechanical and Materials Engineering, North China, University of Technology, Beijing, People’s Republic of China

*Correspondence email: [chzfan@ysu.edu.cn](mailto:chzfan@ysu.edu.cn)

The chemical compositions were examined quantitatively by energy dispersive X-ray spectroscopy (EDX) analysis attached to a Hitachi S-3400N SEM for the purpose of guiding the crystal structure refinement. The examined points are designated in Fig. S1, and the corresponding results are listed in Table S1. The deviation relative to the results of refinement of chemical composition is probably caused by the tilt of the single crystal surface to the incident beam. In addition, the conductive adhesives and glues may also result in the detected impurity elements of carbon and oxygen. For ease of reading, the atomic ratio of the titled phase was calculated and shown in the last column of Table S1.


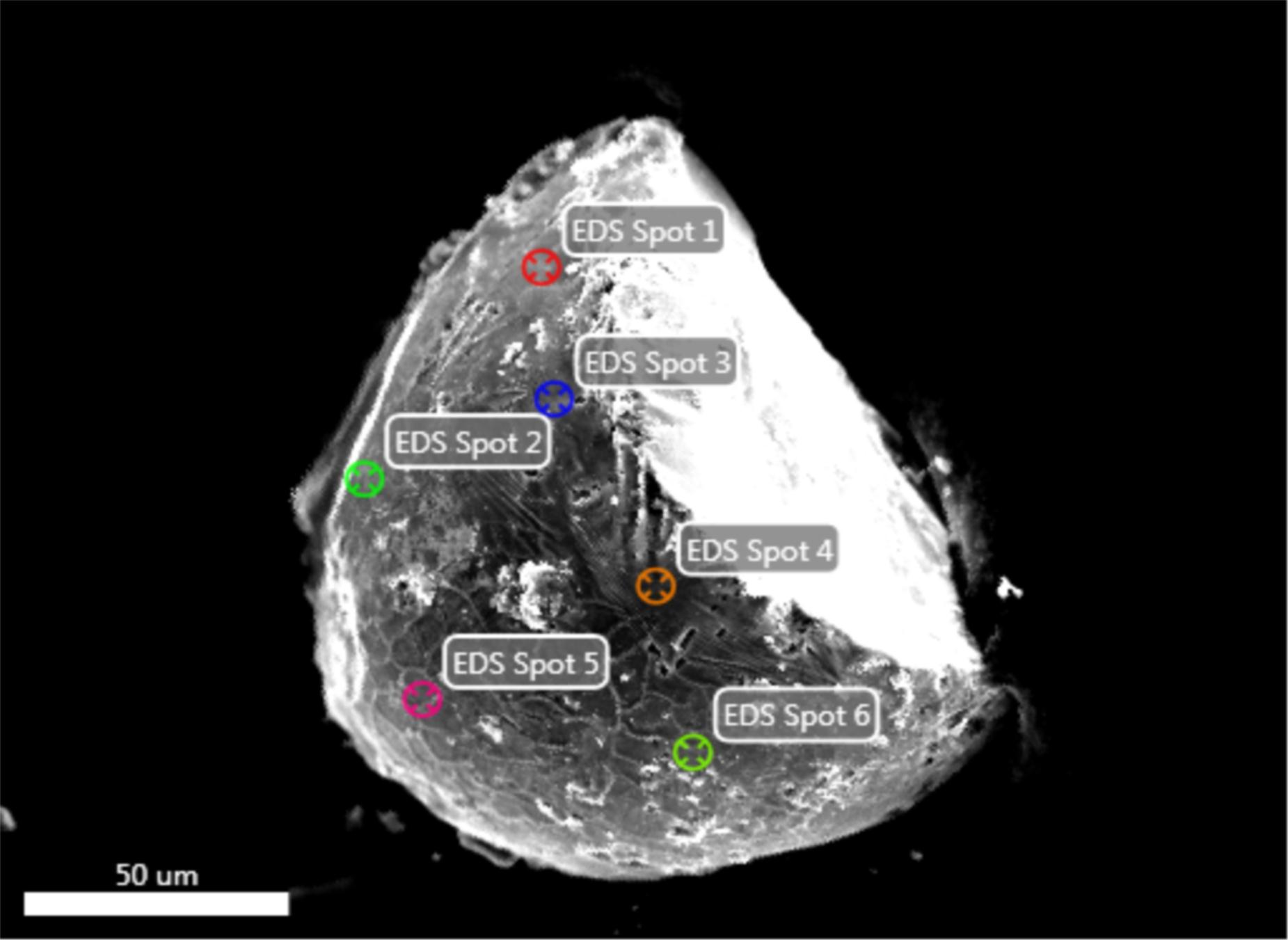


Fig. S1 Single crystal of Al_0.88_Cu_0.94_Fe_0.18_ with selected spots for EDX analysis

**Table S1 EDX results for selected points as designated in Fig. S1**

|  | Element | Weight (%) | Atomic (%) | Error (%) | Al : Fe : Cu |
| --- | --- | --- | --- | --- | --- |
| Spot1 | AlK | 48.07 | 67.69 | 6.89 | 6.48:1:2.09 |
|  | FeK | 15.36 | 10.45 | 3.44 |  |
|  | CuK | 36.57 | 21.86 | 2.91 |  |
| Spot2 | C K | 24.57 | 50.51 | 10.41 | 4.18:1:3.04 |
|  | O K | 7.16 | 11.05 | 9.94 |  |
|  | AlK | 21.53 | 19.71 | 7.00 |  |
|  | FeK | 10.48 | 4.64 | 3.58 |  |
|  | CuK | 36.26 | 14.09 | 2.54 |  |
| Spot3 | AlK | 45.80 | 65.59 | 6.99 | 5.51:1:1.89 |
|  | FeK | 17.19 | 11.90 | 3.29 |  |
|  | CuK | 37.01 | 22.51 | 2.91 |  |
| Spot4 | AlK | 22.34 | 39.83 | 8.26 | 3.52:1:4.31 |
|  | FeK | 13.16 | 11.33 | 3.85 |  |
|  | CuK | 64.51 | 48.84 | 2.45 |  |
| Spot5 | C K | 42.99 | 73.51 | 8.65 | 1:5.46:3.08 |
|  | O K | 5.83 | 7.49 | 10.51 |  |
|  | AlK | 2.62 | 1.99 | 7.77 |  |
|  | FeK | 29.56 | 10.87 | 2.40 |  |
|  | CuK | 18.99 | 6.14 | 3.06 |  |
| Spot6 | C K | 17.85 | 49.68 | 10.23 | 1:2.03:6.06 |
|  | O K | 1.88 | 3.92 | 10.93 |  |
|  | AlK | 4.11 | 5.10 | 8.90 |  |
|  | FeK | 17.34 | 10.38 | 3.22 |  |
|  | CuK | 58.82 | 30.93 | 2.39 |  |

The Al Cu and Fe atoms were refined against different positions as shown in Tab. S2. From the results it is derived that the reported refinement (in bold) is the most suitable one that fit the EDS results.

**Tab. S2** Different choices of refinement and the resulting refined chemical compositions

| location | | compositions | | | | | R1 |
| --- | --- | --- | --- | --- | --- | --- | --- |
| *1a* | *1b* | Al | Fe | | Cu | |  |
| Al | Fe | 0.99984 | 0.99984 | | 0 | | 1.82 |
| Al/Cu | Fe | 0.974454 | 0.99984 | | 0.0253859 | | 1.36 |
| Al/Fe | Fe | 0.965286 | 1.03439 | | 0 | | 1.35 |
| Al | Fe/Al | 1.07073 | | 0.928951 | | 0 | 1.36 |
| Al | Fe/Cu | refinement unstable | | | | |  |
| **Al/Cu** | **Fe/Cu** | **0.882179** | | **0.180301** | | **0.9372** | **1.33** |
| Al/Cu | Fe/Al | refinement unstable | | | | |  |
| Al/Fe | Fe/Cu | 0.862022 | | 0.44042 | | 0.697238 | 1.31 |
